# Evaluating Medical Students’ Perceptions of AI-Assisted Clinical Documentation (CarePilot): Cross-Sectional Study

**DOI:** 10.2196/74198

**Published:** 2026-05-18

**Authors:** Jonathan Bindi, Taylor Jamali, Talia Danze, Iza Zabaneh, Marisa Fat, Joseph Tutera, James Cox

**Affiliations:** 1 Burnett School of Medicine at Texas Christian University, 1651 W Rosedale St, Fort Worth, TX, 76104, United States, 1 9258182512

**Keywords:** artificial intelligence, documentation, satisfaction, medical education, workflow efficiency

## Abstract

**Background:**

Artificial intelligence (AI) is increasingly being integrated into health care to streamline documentation and improve clinician efficiency. AI-powered documentation tools, such as CarePilot, may reduce administrative burdens and help mitigate burnout. However, their usability and perceived value among medical trainees remain underexplored.

**Objective:**

This study aims to evaluate medical students’ perceptions of usability, efficiency, and satisfaction when using an AI-powered documentation system in a simulated clinical setting.

**Methods:**

This cross-sectional study was conducted at the Burnett School of Medicine at Texas Christian University. A total of 44 third- and fourth-year medical students participated in a standardized patient encounter for a headache. Using CarePilot, participants documented patient history, physical examination findings, clinical reasoning, and management decisions. Afterward, they completed a 27-item Likert scale survey assessing ease of use, documentation efficiency, organization, and overall satisfaction.

**Results:**

The majority of respondents rated ease of use, learnability, interface likability, and documentation organization positively (with over 75% of respondents reporting satisfaction for each item). However, a subset reported neutral or dissatisfied opinions regarding overall satisfaction (13/44, 29.5%), citing workflow interruptions and limited functionality that could affect patient interaction.

**Conclusions:**

CarePilot was generally perceived as user-friendly and effective for organizing documentation. Nonetheless, areas for refinement, particularly workflow integration and expanded functionalities, may enhance satisfaction and clinical applicability. These findings inform future design and implementation strategies for AI-powered documentation tools in health care education and beyond.

## Introduction

Artificial intelligence (AI) is transforming health care, particularly in the realm of clinical documentation and workflow optimization. AI-powered tools that transcribe patient interactions and generate structured medical notes have demonstrated potential for reducing administrative burden among health care providers [1,2]. Physicians currently spend an average of 2 hours daily on computer-based documentation, a major contributor to burnout [3].

While AI documentation tools have been studied among residents and attending physicians, little is known about their adoption, usability, and perceived value among medical students. This represents a key knowledge gap, as students’ documentation needs, educational priorities, and workflow constraints differ significantly from those of practicing clinicians. We hypothesized that medical students would report positive experiences with AI documentation assistance, particularly regarding perceived documentation burden.

The medical field has long incorporated dictation systems to reduce manual note-writing time, and AI now enables conversion of dictated content into structured, physician-style documentation. Some tools, such as CarePilot, also predict medical coding from recorded encounters. Although research suggests AI will likely become a routine part of multiple medical specialties [4], the extent to which medical students will adopt and benefit from these systems remains unclear. Existing literature includes a mix of opinion pieces, preliminary studies, and peer-reviewed trials [5-7], but few have systematically examined the student population in a controlled setting.

Recent work has shown that AI-assisted clinical documentation can improve efficiency, accuracy, and even suggest clinical phenotypes and diagnoses [4]. However, generalizing these findings to medical students requires caution. Students operate in supervised educational contexts with lower patient loads and different performance expectations, making their experiences with AI documentation tools potentially distinct from those of fully licensed physicians.

This study aims to address this gap by assessing initial usability and satisfaction with an AI documentation tool among third- and fourth-year medical students in a controlled, simulation-based environment. This exploratory evaluation is not intended to determine the tool’s effectiveness in real clinical practice but rather to establish a baseline understanding of student perceptions and identify potential facilitators and barriers to adoption.

## Methods

### Ethical Considerations

The study protocol was approved by the Institutional Review Board of Texas Christian University #2024-119, and all participants provided written informed consent before participation.

Survey responses were collected anonymously, and no personally identifiable information was recorded or linked to participant data. All data were stored in a secure, password-protected database accessible only to study investigators.

The postencounter survey was administered electronically using Qualtrics as a closed, web-based questionnaire accessible only to invited participants. Participation was voluntary, and informed consent was obtained prior to survey access. The survey was administered immediately following the simulated encounter and could be completed only once per participant. Completeness checks were not enforced, and participants were not required to answer all questions to submit the survey. Incomplete responses were included in analyses using available data only.

Participants did not receive financial compensation for participation in this study.

### Study Design and Setting

This cross-sectional study was conducted at the Burnett School of Medicine at Texas Christian University to evaluate medical students’ experiences during a simulated clinical encounter using CarePilot services.

### Participants

Medical students enrolled in the clinical phase of their training (eg, third- and fourth-year students) were invited to participate via institutional email announcements and classroom announcements. Inclusion criteria required that students be actively participating in clinical rotations at the time of the study. Students with prior extensive experience using the CarePilot system (defined as more than two standardized patient encounters or more than one full clinical rotation involving CarePilot) or who had recently participated in similar simulation exercises were excluded to minimize potential bias.

### Simulation Scenario

A standardized patient encounter was developed around a chief complaint of a headache. The scenario was designed in consultation with clinical faculty and simulation experts to ensure that key elements of history-taking, physical examination, and initial management were addressed uniformly across all participants. Standardized instructions and case materials were provided before the encounter to ensure consistency in presentation.

### Use of CarePilot

During the simulation, each participant was required to use the CarePilot system to document their patient encounter. CarePilot served as the electronic health record (EHR) platform for this exercise, allowing participants to capture encounter details, generate structured notes aligned to common templates, and record reasoning and management decisions. The system was configured to mimic the workflow of a typical clinical encounter, and technical support was available throughout the session to address any issues related to system use.

### Procedure

Each simulation session followed a standardized timeline:

1. Pre-encounter briefing: Participants received a brief orientation outlining the objectives of the simulation and instructions for using CarePilot.
2. Simulated encounter: Participants engaged in a clinical encounter with a standardized patient presenting with a chief complaint of a headache. During the encounter, participants documented their findings and management decisions using CarePilot.
3. Postencounter survey: Immediately following the simulation, participants were asked to complete a 27-question survey. The survey was administered electronically and took approximately 2 to 5 minutes to complete.

### Survey Instrument

The 27-item survey instrument was developed through a structured process informed by a targeted review of literature on AI-assisted documentation systems and usability evaluation in health care settings. Items were designed to assess domains relevant to medical student experiences with AI documentation tools, including perceived ease of use, efficiency, workflow integration, documentation organization, perceived usefulness, and overall satisfaction.

To support content validity, the initial item set was reviewed by faculty with expertise in medical education and health technology implementation. Reviewers evaluated clarity, redundancy, and alignment with study objectives. Revisions were made iteratively based on this feedback.

Before study deployment, the survey was pilot-tested with a small group of medical students and faculty members not included in the final cohort. Participants provided feedback regarding clarity and comprehensiveness, and minor wording adjustments were made accordingly. Although the instrument was not derived from a previously validated scale, these steps were undertaken to enhance face and content validity within the exploratory scope of this study.

### Data Collection and Analysis

Survey responses were collected electronically via Qualtrics and exported into a secure database for analysis. Descriptive statistics were calculated for demographic variables and Likert scale responses. Analyses were conducted using Excel, Version 16.44 (Microsoft Corporation).

The survey instrument was designed to assess multiple distinct domains of user perception, including usability, workflow impact, perceived clinical value, and overall satisfaction. Because the items were intended to represent related but conceptually distinct constructs, responses were analyzed individually using descriptive statistics rather than combined into composite or subscale scores, consistent with recommended approaches for interpreting individual Likert-type items as ordinal data [8]. Accordingly, measures of internal consistency (eg, Cronbach α) were not calculated, as the instrument was not developed to function as a unidimensional psychometric scale.

## Results

### Medical Student Satisfaction With the Documentation System

**Figure 1.**
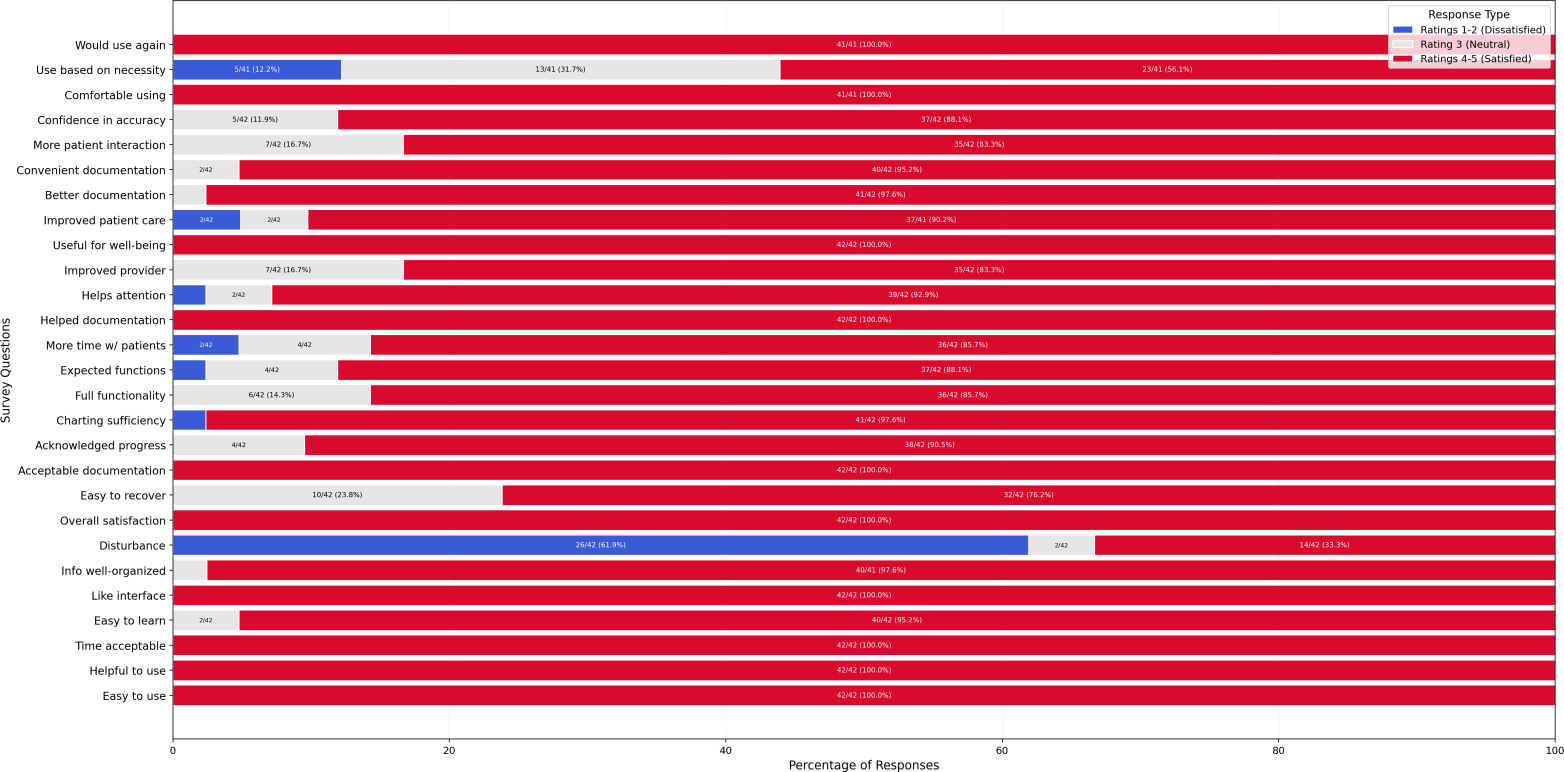
Likert scale breakdown of responses, displaying satisfaction, neutrality, and dissatisfaction across survey questions.

A Likert scale–based survey was conducted to assess user satisfaction with the documentation system. The distribution of responses across survey items is shown in Figure 1. Item-level descriptive statistics, including the mean and SD for each question, are presented in Table 1.

**Table 1.** Item-level descriptive statistics for the 27 survey questions assessing medical student perceptions of CarePilot using a Likert scale from 1 (strongly disagree) to 5 (strongly agree).

Survey item	Scores, mean (SD)
Easy to use	4.90 (0.30)
Helpful to use	4.90 (0.33)
Time acceptable	4.80 (0.40)
Easy to learn	4.70 (0.55)
Like interface	4.70 (0.45)
Information well-organized	4.90 (0.42)
Disturbance	2.60 (1.77)
Overall satisfaction	4.90 (0.35)
Easy to recover	4.30 (0.85)
Acceptable documentation	4.80 (0.40)
Acknowledged progress	4.50 (0.67)
Charting sufficiency	4.70 (0.59)
Full functionality	4.50 (0.74)
Expected functions	4.60 (0.77)
More time with patients	4.40 (0.93)
Helped documentation	4.80 (0.38)
Helps attention	4.60 (0.80)
Improved provider	4.50 (0.77)
Useful for well-being	4.80 (0.42)
Improved patient care	4.60 (0.81)
Better documentation	4.80 (0.48)
Convenient documentation	4.80 (0.52)
More patient interaction	4.50 (0.77)
Confidence in accuracy	4.50 (0.71)
Comfortable using	4.90 (0.36)
Use based on necessity	3.90 (1.17)
Would use again	4.90 (0.30)

Across most survey questions, responses were clustered toward the upper end of the Likert scale, indicating generally favorable perceptions of usability and efficiency. The highest mean scores were observed for ease of use, helpfulness, information organization, and likelihood of future use. The “disturbance” item demonstrated greater variability; however, its lower mean score reflected overall disagreement with the statement that the system disrupted the clinical encounter. In contrast, “use based on necessity” showed a comparatively lower mean, suggesting some heterogeneity in perceived value and voluntary adoption.

### Overall Satisfaction and Key Findings

Across most survey questions, the proportion of ratings in the 4 to 5 category exceeded 75%, indicating generally favorable perceptions of the system’s usability, documentation efficiency, and functional adequacy. The highest mean scores were observed for ease of use (4.90, SD 0.30; 4‐5 ratings: 42/44, 95.5%), helpful to use (4.90, SD 0.33; 4‐5 ratings: 42/44, 95.5%), information organization (4.90, SD 0.42; 4‐5 ratings: 40/44, 90.9%), comfortable using the system (4.90, SD 0.36; 4‐5 ratings: 41/44, 93.2%), and would use again (4.90, SD 0.30; 4‐5 ratings: 41/44, 93.2%).

Items reflecting potential limitations or mixed perceptions exhibited greater heterogeneity. The reverse-coded “disturbance” item had a mean of 2.60 (SD 1.77). Because lower scores indicate less perceived disturbance (ie, disagreement that the system disrupted the clinical encounter), the mean suggests minimal disruption on average, while the large SD indicates substantial variability in perceived workflow impact across participants. Consistent with this pattern of heterogeneity, “use based on necessity” showed a comparatively lower mean (3.90, SD 1.17), suggesting that some respondents perceived system use as driven more by necessity than interest. For items assessing whether the system met expectations and influenced patient-facing time, a minority of respondents selected neutral or dissatisfied ratings (approximately 15%): expected functions (neutral/dissatisfied: 5/44, 11.4%) and more time with patients (neutral/dissatisfied: 6/44, 13.6%). Overall, usability perceptions and reuse intention were consistently high, whereas variability was more evident in perceived workflow integration and the extent to which use was driven by necessity.

### Implications for Future Improvements

While the survey indicates strong satisfaction levels with most aspects of the system, the identified areas of concern suggest potential avenues for refinement. Addressing usability concerns related to workflow disturbance, functionality expectations, and overall satisfaction could further enhance adoption rates and long-term engagement with the system.

## Discussion

In this simulation-based exploratory study, medical students reported overall positive perceptions of the CarePilot system, particularly regarding ease of use, learnability, and documentation efficiency. However, some respondents noted challenges related to workflow disturbance and functional expectations, highlighting areas for potential improvement. While these findings suggest that CarePilot can support efficient note generation and reduce cognitive effort in an educational simulation, the extent to which these benefits will translate to real-world practice remains uncertain and should be investigated further rather than interpreted as definitive outcomes.

Several studies have shown that AI-powered documentation tools can improve workflow efficiency and reduce administrative burden in active clinical settings. Previous studies have reported significant reductions in documentation time and after-hours charting in multisite clinical trials [3]. Others found that ambient AI scribes shortened note completion times without compromising quality [9]. Shah et al [10] and Alboksmaty et al [11] further demonstrated improvements in clinician satisfaction and reductions in burnout scores in primary care and outpatient contexts. Our results are consistent with these observations in that users reported high satisfaction with efficiency and usability. However, unlike those real-world studies, our work was conducted in a simulation with medical students who do not face the same time pressures, billing requirements, or patient volumes as practicing clinicians. Because medical students do not shoulder full clinical responsibility, their perceptions may not generalize to practicing clinicians. This difference in user profile and environment should temper direct comparisons, though the strong usability signals we observed suggest potential for similar benefits if implemented with appropriate adaptation.

Not all evaluations of AI documentation systems have been uniformly positive. This pattern is consistent with prior work showing that although clinicians value generative AI for improving documentation efficiency, they remain cautious about reliability, automation bias, medico-legal risk, and the need for continued clinician supervision [12]. Even when efficiency gains are realized, challenges such as workflow interruptions, system lag, and imperfect transcription accuracy can undermine adoption [13]. Similar concerns were raised by participants in our study, with approximately 30% reporting neutral or negative overall satisfaction and identifying workflow interruptions as a key limitation. These disturbances may have stemmed from a combination of factors, including occasional system latency, the need for mid-encounter corrections, and an interface design that required shifting attention away from the patient. Identifying and addressing such sources could improve the fit into the natural flow of a clinical encounter. The alignment between these reported barriers in both simulated and real clinical contexts reinforces the importance of optimizing speed, accuracy, and EHR integration before widespread implementation.

Our findings also align with the broader literature on clinician burnout and documentation burden. While AI systems can meaningfully reduce charting time, their success depends on high usability, minimal cognitive load, and robust technical support [2]. In our cohort, the intuitive interface and ease of learning likely contributed to positive perceptions, mirroring the importance of these factors in real-world adoption. However, claims about enhancing the learning experience, reducing cognitive load, or mitigating burnout should be framed as potential benefits that merit further research rather than as direct conclusions from the current study.

In addition to efficiency, AI-assisted documentation has implications for clinical communication and patient engagement. When well-integrated, such systems can free clinicians to spend more time face-to-face with patients [11]. Conversely, poor integration risks distracting from patient interactions. Our results point to a similar tension: while many participants valued the time savings, some noted that the need to monitor and edit the system’s output could detract from patient focus. Future iterations of CarePilot could incorporate features such as real-time summarization prompts or patient-facing summaries to strengthen this aspect while maintaining accuracy under clinician supervision.

Successful adoption will also require targeted training and adaptation to the specific needs of different clinical environments. Studies across diverse specialties have shown that familiarity with the system, iterative user feedback, and integration into authentic workflows are critical for sustained use. Our results, though limited to an educational simulation, underscore these same principles. Additionally, the potential influence of self-selection bias and the Hawthorne effect should be acknowledged, as participants who chose to enroll and knew they were being observed may have reported more favorable impressions.

While our survey was designed through a targeted literature review and expert consultation, it was not directly adapted from established, validated usability instruments such as the System Usability Scale or the technology acceptance model. Instead, items were tailored to capture domains relevant to medical student experiences with AI-assisted documentation, including perceived efficiency, ease of use, and workflow integration. The instrument underwent face and content validation through review by faculty with expertise in medical education and health technology, and was pilot-tested with a small cohort of medical students before implementation. Although this process supported clarity and relevance of items for our specific context, the lack of use of a formally validated instrument may limit comparability with other studies. Future work could incorporate or adapt validated frameworks to enhance psychometric rigor and facilitate cross-study benchmarking.

Additionally, our findings highlight overall positive perceptions of CarePilot’s usability, efficiency, and organization. The interpretation of these results must be considered in light of alternative explanations. Factors such as prior exposure to EHR systems, individual comfort with technology, and simulation-specific conditions (eg, absence of real clinical time pressures, controlled patient presentations) may have influenced participants’ responses. Additionally, we did not collect demographic variables such as gender, age, or socioeconomic status, which could reveal important subgroup differences in perceptions of usability and satisfaction. Incorporating these variables in future research would allow for a more nuanced understanding of how personal and contextual factors influence technology adoption among medical trainees. Addressing these considerations will help ensure that implementation strategies are tailored to diverse user needs and training environments.

This study has several limitations that should be considered when interpreting the findings. It was conducted in a simulated clinical setting, which may not fully reflect the complexities and pressures of real-world environments. The controlled nature of the simulation and the use of medical students may limit generalizability to practicing clinicians. The sample size was relatively small and drawn from a single institution, further constraining external validity. Moreover, self-selection bias and the Hawthorne effect may have influenced responses.

Despite these limitations, our findings suggest that AI-assisted documentation tools such as CarePilot hold promise for improving usability and efficiency, with potential downstream benefits for clinician workflow, education, and patient engagement. These potential benefits should be validated in larger, more diverse, real-world studies. Future research should explore integration with existing EHR platforms, enhance real-time data capture, minimize workflow disturbances, and assess long-term effects on clinician efficiency, burnout, and patient outcomes. Addressing these challenges will be essential to ensuring that AI-driven documentation solutions contribute positively to clinical practice while preserving the quality of physician-patient interactions.

## Abbreviations

AI: artificial intelligence
EHR: electronic health record

## References

[1] Bundy H, Gerhart J, Baek S (2024). Can the administrative loads of physicians be alleviated by AI-facilitated clinical documentation?. J Gen Intern Med.

[2] Bracken A, Reilly C, Feeley A, Sheehan E, Merghani K, Feeley I (2025). Artificial intelligence (AI) - powered documentation systems in healthcare: a systematic review. J Med Syst.

[3] Stults CD, Deng S, Martinez MC (2025). Evaluation of an ambient artificial intelligence documentation platform for clinicians. JAMA Netw Open.

[4] Perkins SW, Muste JC, Alam T, Singh RP (2024). Improving clinical documentation with artificial intelligence: a systematic review. Perspect Health Inf Manag.

[5] Ng JJW, Wang E, Zhou X (2025). Evaluating the performance of artificial intelligence-based speech recognition for clinical documentation: a systematic review. BMC Med Inform Decis Mak.

[6] Barak-Corren Y, Wolf R, Rozenblum R (2024). Harnessing the power of generative AI for clinical summaries: perspectives from emergency physicians. Ann Emerg Med.

[7] Poon EG, Lemak CH, Rojas JC, Guptill J, Classen D (2025). Adoption of artificial intelligence in healthcare: survey of health system priorities, successes, and challenges. J Am Med Inform Assoc.

[8] Sullivan GM, Artino AR (2013). Analyzing and interpreting data from Likert-type scales. J Grad Med Educ.

[9] Ma SP, Liang AS, Shah SJ (2025). Ambient artificial intelligence scribes: utilization and impact on documentation time. J Am Med Inform Assoc.

[10] Shah SJ, Devon-Sand A, Ma SP (2025). Ambient artificial intelligence scribes: physician burnout and perspectives on usability and documentation burden. J Am Med Inform Assoc.

[11] Alboksmaty A, Aldakhil R, Hayhoe BWJ, Ashrafian H, Darzi A, Neves AL (2025). The impact of using AI-powered voice-to-text technology for clinical documentation on quality of care in primary care and outpatient settings: a systematic review. EBioMedicine.

[12] Fraile Navarro D, Kocaballi AB, Berkovsky S (2025). Understanding clinician perceptions of GenAI: a mixed methods analysis of clinical documentation tasks. J Med Syst.

[13] Sarraf B, Ghasempour A (2025). Impact of artificial intelligence on electronic health record-related burnouts among healthcare professionals: systematic review. Front Public Health.

